# Cationic Calix[4]arene Vectors to Efficiently Deliver AntimiRNA Peptide Nucleic Acids (PNAs) and miRNA Mimics

**DOI:** 10.3390/pharmaceutics15082121

**Published:** 2023-08-10

**Authors:** Jessica Gasparello, Chiara Papi, Matteo Zurlo, Stefano Volpi, Roberto Gambari, Roberto Corradini, Alessandro Casnati, Francesco Sansone, Alessia Finotti

**Affiliations:** 1 Section of Biochemistry and Molecular Biology, Department of Life Sciences and Biotechnology, University of Ferrara, 44121 Ferrara, Italy; gspjsc@unife.it (J.G.); chiara.papi@unife.it (C.P.); matteo.zurlo@unife.it (M.Z.); gam@unife.it (R.G.); 2 Department of Chemistry, Life Sciences and Environmental Sustainability, University of Parma, 43124 Parma, Italy; stefano.volpi@unipr.it (S.V.); roberto.corradini@unipr.it (R.C.); alessandro.casnati@unipr.it (A.C.)

**Keywords:** delivery, calix[4]arene, Peptide Nucleic Acids, microRNA, miRNA-targeting, microRNA-mimics, premiRNA, antimiRNA

## Abstract

One of the most appealing approaches for regulating gene expression, named the “microRNA therapeutic” method, is based on the regulation of the activity of microRNAs (miRNAs), the intracellular levels of which are dysregulated in many diseases, including cancer. This can be achieved by miRNA inhibition with antimiRNA molecules in the case of overexpressed microRNAs, or by using miRNA-mimics to restore downregulated microRNAs that are associated with the target disease. The development of new efficient, low-toxic, and targeted vectors of such molecules represents a key topic in the field of the pharmacological modulation of microRNAs. We compared the delivery efficiency of a small library of cationic calix[4]arene vectors complexed with fluorescent antimiRNA molecules (Peptide Nucleic Acids, PNAs), pre-miRNA (microRNA precursors), and mature microRNAs, in glioma- and colon-cancer cellular models. The transfection was assayed by cytofluorimetry, cell imaging assays, and RT-qPCR. The calix[4]arene-based vectors were shown to be powerful tools to facilitate the uptake of both neutral (PNAs) and negatively charged (pre-miRNAs and mature microRNAs) molecules showing low toxicity in transfected cells and ability to compete with commercially available vectors in terms of delivery efficiency. These results could be of great interest to validate microRNA therapeutics approaches for future application in personalized treatment and precision medicine.

## 1. Introduction

MicroRNA (miRNA) therapeutics [1,2] is one of the most appealing and novel approaches proposed for several human pathologies [1,2,3,4,5]. Its rationale is based on the fact that miRNAs, small non-coding RNAs, that are 20–23 nucleotides long, are able to regulate gene expression through binding with complementary mRNA sequences [3,4,5]. A key finding that sustains the concept of microRNA therapeutics is that miRNA dysregulation has been demonstrated to be associated with many severe diseases [6,7,8,9,10]. MicroRNA therapeutics consists of (a) miRNA inhibition by antimiRNA molecules (targeting up-regulated miRNAs that cause pathological conditions) and (b) miRNA replacement using miRNA mimics (in the case that the activity of down-regulated miRNAs should be restored) [11,12,13]. Despite most of these studies still being at the pre-clinical level, some miRNA therapeutics have reached clinical development, giving promising results in the treatment of viral infections (e.g., hepatitis) and cancer [14,15,16].

An example of validated antimiRNA molecules is antisense oligonucleotides (ASOs), which are often submitted to a series of chemical modifications that improve their stability, pharmacokinetic features, and recognition of target miRNAs [17,18]. For example, Miravirsen (SPC3649, Santaris Pharma), a molecule that has completed phase 2 clinical trials for the treatment of chronic hepatitis C virus (HCV) infection [19,20], is a locked nucleic acid (LNA) interspaced throughout a DNA phosphorothioate sequence that binds to mature miR-122. A series of pre-clinical studies indicated the high potential of Peptide Nucleic Acids (PNAs) as antimiRNA molecules which are able to perform miRNA inhibition by steric blocking strategies [21]. PNAs are particularly suited for this purpose thanks to their neutral, pseudopeptide backbone that imparts them with inertness towards nuclease and peptidase enzymes, and a remarkable affinity and selectivity for complementary targets [22,23]. These features have been exploited in cancer cellular models (i.e., colorectal cancer, glioma, glioblastoma, breast cancer, leukemia) [24,25,26,27,28,29] and genetic diseases (i.e., cystic fibrosis) [30]. PNAs were even found to be effective for some in vivo application [31,32]. For example, an antimir-155 PNA was used to inhibit tumor growth in a murine model of lymphoma, providing a promising candidate for the translation of this type of artificial oligonucleotides to clinics [32,33].

As regards miRNA mimics, MRX34 (NCT01829971) is a synthetic, double-stranded miR-34a mimic encapsulated in liposomal nanoparticles, and currently being studied in a phase 1 clinical trial against cancer [34]. The rationale of mimicking miR-34a is based on the evidence that it behaves as a tumor-suppressor by targeting more than 30 oncoprotein-coding mRNAs, but it is downregulated in several solid tumors. Another miRNA mimic (TargomiRs, NCT02369198, Phase 1) is used to treat Malignant Pleural Mesothelioma (MPM) and Non-Small Cell Lung Cancer (NSLC) [35]. TagomiRs is a double-stranded, synthetic, and RNA-based molecule that mimics miR-15 and miR-107, which are frequently dysregulated in lung cancer [10,35]. These are only a few examples of molecules involved in a clinical trial or in advanced pre-clinical evaluation, while several reviews excellently summarize the landscape of miRNA-based therapies [36,37,38,39,40].

In such a context, developing new efficient, low-toxic and targeted tools to deliver this kind of molecule represents a key topic in the field of the pharmacological modulation of microRNAs [41]. Non-viral delivery methods for DNA are generally compatible with miRNA therapeutics, especially those bearing a negative sugar phosphate or phosphorothioate backbone (e.g., natural or modified RNAs, LNAs). The chemical modification of oligonucleotides, generally exploited to modulate the stability and pharmacokinetics of these molecules, can also improve their cellular uptake by affecting for example their interaction with plasma proteins [41]. This approach also includes their covalent conjugation with carrier molecules, such as GalNac moieties [41,42,43], peptides [44,45,46,47], antibodies [48,49], and lipid units [50,51,52], that, in other fields of oligonucleotide therapeutics have proven effective for clinical applications. Lipid nanoparticles and liposomes, forming the so-called lipoplexes and based on a mixture of cationic (i.e., DOTAP, DOTMA, DOGS), neutral, and ionizable lipids, represent another rather mature technology [41,53]. An interesting example of polymeric vectors is represented by blended polylactic co-glycolic acid (PLGA)/poly-β-aminoester (PBAE) nanoparticles, which were reported to combine a high loading of plasmid DNAs with a good biocompatibility [54]. Cationic polymers and dendrimers such as PEI and PAMAM could also be an alternative, even if their high toxicity and low efficiency can be only partly mitigated by their combination with PEG chains, cell penetrating peptides, or fatty acids [41,55]. These formulations usually imply the use of adjuvants and/or additives to improve cell membrane penetration and release. The intracellular delivery of PNAs requires instead dedicated strategies, since their neutral backbones generally impair their loading into the vectors developed for natural nucleic acids and their negatively charged analogs [56,57,58]. An effective strategy to solve this issue is the modification of the PNA structure, to promote their direct uptake or their inclusion into proper delivery systems. Their conjugation with cell penetrating peptides has been widely explored, with a particular focus on polyarginine chains or tumor-homing sequences [31,41,59,60,61,62,63]. Covalently linked anionic peptides or lipophilic moieties have been, instead, exploited to promote their inclusion into lipid nanoparticles [64,65]. Despite these approaches looking promising for several applications, PNA modification is often labor intensive and sometimes results in significant cytotoxicity, thus promoting the development of nanoscaled carriers that do not require a covalent conjugation to these molecules [65,66,67]. PNAs can be loaded into cationic nanoparticles prior to their combination with a partly complementary DNA, from which the PNA strand can be released upon the recognition of a fully complementary target [66,67,68]. Other types of carriers, such as PLGA [39,69,70,71,72,73,74], silica [75,76], and silicon nanoparticles [77,78,79,80], are instead suitable for loading naked PNAs with high efficiency, resulting, in some cases, in suitability for in vivo applications. However, these systems often rely on additional building blocks (i.e., peptides, PEG, or lipid chains) to provide an optimal delivery, negatively counterbalancing the possibility of using unmodified PNAs with the need for multicomponent materials with challenging assembly protocols [65,66,67]. 

**Figure 1:**
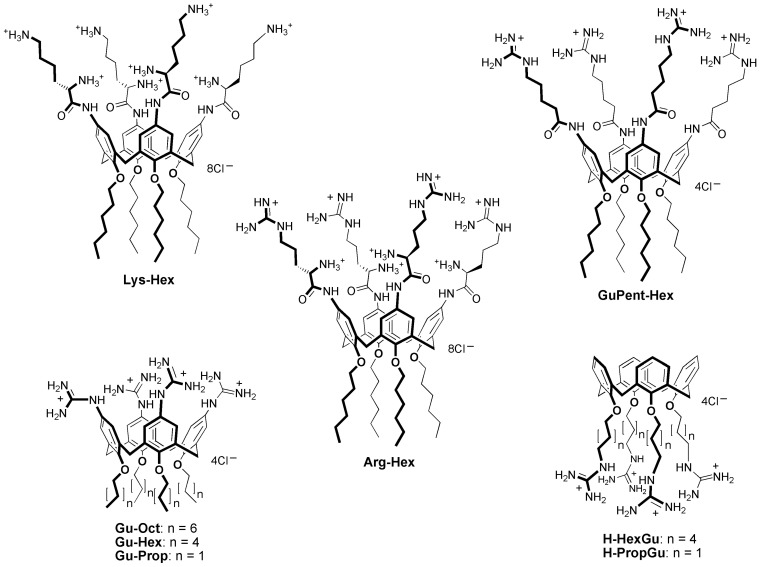
Structures of the calix[4]arene vectors. To aid the reader in identifying the structure of the calixarene vectors, we introduced in the figure and in the text a running nomenclature indicating, at the left of the dash, the residue at the upper rim (aromatic nuclei) and, at the right of the dash, the residue at the lower rim (phenolic oxygens).

Hence, although the state of the art for miRNA therapeutics delivery is constantly improving, the research for vectors characterized by negligible toxicity, high efficiency, high loading capacity, selective targeting, propensity to cargo release after cell uptake, and ease of formulation is still an open challenge for the scientists engaged in the field. In this context, we recently proposed a macrocyclic, multivalent tetra-L-argininocalix[4]arene (**Arg-Hex**, Figure 1) which was initially designed for DNA delivery [81], but was subsequently also found to be able to transport both RNA-based molecules (e.g., mature miRNAs, premiRNAs and antimiRNA oligonucleotides) [82] and antimiRNA PNAs [83,84] into cells with a high delivery efficiency, a low cytotoxicity, while preserving the biological activity of the delivered cargos.

In the present work, we have evaluated a small library of calix[4]arene vectors which have distinct structural modifications compared to **Arg-Hex**, considered as ‘reference compound’, in order to test their ability to deliver (a) antimiRNA PNAs and (b) microRNA mimics, with the aim of correlating their delivery efficiency with their chemical structure and ability of making available a family of potential delivery systems with tunable features. Our interest in calix[4]arenes is based on recent data which demonstrate that this class of vectors displays very low levels of toxicity compared with “gold-standard carriers” [85]. In plasmid delivery, they were also demonstrated to be much more efficient than the corresponding non-macrocyclic analogs, evidencing a dramatic impact on their activity by the arrangement of the cationic head groups in clusters on the calixarene platform [81,86,87,88]. We have already reported that **Arg-Hex**, the lead compound from this class of vectors, exhibited low toxicity and no inhibitory effects on the growth of treated cells, unlike the gold-standard lipofectamine, which caused the inhibition of cell growth, as expected from the results of several reports. More importantly, a PNA against microRNA miR-221-3p fully maintained its biological activity and specificity in inhibiting the target miR-221-3p-mediated effects (i.e., induction of apoptosis) [83,84]. These important issues have been recently discussed in Finotti et al. [84]. The preservation of the biological activity has been fully confirmed in the case of calix[4]arene-delivered pre-miRNA molecules [82]. This study has demonstrated that the biological activity of transfected pre-miRNA (i.e., the induction of apoptosis by premiR-124 in U251 cells) is fully maintained after calix[4]arene-assisted delivery.

The uptake by target cells is the first step to be studied in characterizing the possible biotechnology and biomedical applications of vectors proposed for the delivery of bioactive molecules. Therefore, the focus of the present study was to characterize the structures of the calix[4]arene-class of delivery systems in respect to the uptake of PNAs and pre-miRNA molecules.

## 2. Materials and Methods

### 2.1. Calix[4]arene Vectors Synthesis and Resuspension

Calix[4]arene vectors were synthesized as reported in the literature [81,86,87,88,89,90] and briefly summarized in Supplementary Methods S1. Vectors were then resuspended in a solution of water:ethanol:DMSO (2:2:1) or water:DMSO (1:1) in sterile conditions. After the solubilization, they were stored at −20 °C until use.

### 2.2. Cell Lines and Culture Conditions

The human glioma U251 and the human colon cancer cell line HT29 were cultured, respectively, in DMEM-high glucose, then added with L-glutamine, and in RPMI-1640 medium (Euroclone) as described elsewhere [32]. All cell cultures were carried out at 37 °C in a humidified atmosphere and in the presence of 10% Fetal Bovine Serum (FBS) (Biowest, Nuaille, France). Detailed culture conditions are reported in Supplementary Methods S3. 

### 2.3. Transfection Procedures

The same transfection procedure was used for PNA, PremiRNA (miRNA precursor) and mature miRNA delivery. In brief, a mixture containing culture medium (DMEM or RPMI for U251 and HT-29, respectively), calix[4]arene vector at final concentration of 2.5 µM, and premiRNA, mature miRNA, or antimiRNA PNA was prepared and incubated for 20 min at room temperature, in the absence of serum in order to avoid serum-dependent effects. At the end of the incubation, 10% (*v*/*v*) of FBS was added. Cell culture medium was removed and replaced with the transfection mixture. Transfection mixture was maintained in contact with cells for the duration of transfection. A PNA with the same sequence but functionalized with a polyarginine (R8) was used as positive control for PNA transfection experiments: cells were treated for 24 h with 1.5 µM of R8-PNA. Lipofectamine RNAiMAX, a commercial formulation of cationic liposomes, was used as positive control for mature miRNA transfection experiments, following manufacturer’s instructions. Briefly, Lipofectamine RNAiMAX was diluted in Opti-MEM I Reduced Serum Medium (Gibco, Thermo Fisher Scientific, Waltham, MA, USA), mature miRNA was added, and the mixture was incubated for 5 min at room temperature and then transferred to cells. PremiRNA (premiR-221, PM10337) was purchased from Ambion (Thermo Fisher Scientific, Waltham, MA, USA) and was employed at final concentration of 100 nM. Fluorescein-labelled, mature miRNA 210-3p (5′-Fl-CUGUGCGUGUGACAGCGGCUGA-3′) was purchased from IDT (Integrated DNA Technology) and employed at final concentration of 100 nM. Fluorescent naked antimiR-221-PNA (FAM-O-AAACCCAGCAGACAATGT) was synthesized as reported elsewhere and used at final concentration of 1.5 µM [33]. For FACS analyses and RT-qPCR assays, 12 wells plates were used in a total volume of 0.4 mL. In each well 120,000 U251 cells and 100,000 HT29 cells were seeded.

### 2.4. FACS Analysis

Uptake of the mature fluorescent miRNA and fluorescent naked PNA was evaluated using FACS Canto II (BD, Franklin Lakes, NJ, USA). Twenty-four hours after the transfection, cells were detached, washed twice with Dulbecco’s phosphate-buffered saline (DPBS) 1×, resuspended in 200 μL of DPBS 1×, and analyzed by FACS analysis for fluorescein isothiocyanate (FITC) fluorescence. For each sample, 10,000 events were acquired, and data analysis was performed using FACSDiva software Ver. 8.0 (BD, Franklin Lakes, NJ, USA).

### 2.5. RNA Extraction

After the collection by centrifugation at 1500 rpm for 5 min at 4 °C, cells were lysed with Tri-Reagent (Sigma Aldrich, St. Louis, MO, USA) following manufacturer’s instructions. Isolated RNA was washed once with cold 75% ethanol, air-dried, and dissolved in nuclease-free water before use.

### 2.6. Quantification of miRNAs

The miRNAs quantification was performed by CFX96 touch real-time PCR detection system (Bio-Rad, Hercules, CA, USA), using the two-step protocol of the TaqMan MicroRNA Assays (Applied Biosystems Thermo Fisher Scientific, Foster City, CA, USA), which employs a target-specific stem–loop primer during cDNA synthesis to produce a template for real-time PCR. In the first step for the cDNA production, starting from total RNA, we used a miR-221 stem-loop primer, included in the TaqMan microRNA reverse transcription kit (Applied Biosystems) (ID: 000524), specific for cDNA production from the mature miRNA sequence: 5′-AGCUACAUUGUCUGCUGGGUUUC-3′. The obtained cDNA was the template for the real-time PCR, following the protocol described by the manufacturer (15 s at 95 °C and 15 s at 60 °C, for 40 cycles). Comparative cycle threshold (ΔΔCT) method was employed to calculate miR-221-3p fold change, using hsa-let-7c (hsa-let-7c, ID: 000379) as endogenous controls.

### 2.7. MTT Assay

To verify possible cytotoxic effects of the calix[4]arene vectors at the concentrations used in the present study, MTT assay was performed. Cells were seeded in a 96-multiwell plate (0.2 mL final volume) and transfected with calix[4]arene vectors. Cells were incubated at 37 °C for 24 h (short term protocol) or 72 h (long term protocol) and at the end of the incubation period, MTT was added to each well at a final concentration of 0.5 mg/mL. Following 3 h of incubation at 37 °C, the medium was discarded and dimethyl sulfoxide (DMSO) was added; the plate was stirred for 30 min to fully dissolve the formazan crystals formed at the bottom of the wells. The absorbance was measured at 570 nm using the SUNRISE microplate reader (Tecan Group, Ltd., Männedorf, Switzerland).

### 2.8. Cell Imaging

The internalization of calix[4]arene-delivered cargos was evaluated using BioStation IM (Nikon, Minato, Tokyo, Japan), thanks to the use of fluorescein-labelled molecules. After 24 h contact, transfection mixture was removed and cells were treated with Hoechst 33342 dye, at final concentration of 0.1 µg/mL, to identify nucleus. Images were taken using DAPI filter (461 nm) to visualize nuclei and 530 nm filter to visualize fluorescein conjugate molecules (mature miRNA and PNA). In these experiments, 4-well Biostation plates were used (surface: 0.8 cm^2^ each) and the experiments were started using 60% and 40% confluency U251 and HT29 cells, respectively. Three different magnifications were employed: ×20, ×40, and ×80.

### 2.9. Statistical Analysis

All the data were normally distributed and presented as mean ± S.E.M. Statistical differences between groups were compared using a paired *t*-test or a one-way repeated-measures ANOVA (ANalyses of VAriance between groups) followed by LSD post-hoc tests. Statistical differences were considered significant when *p* < 0.05 (*) and highly significant when *p* < 0.01 (**).

## 3. Results

### 3.1. Design of the Calix[4]arene Vectors

The interesting and encouraging results obtained with derivative **Arg-Hex** in delivering both PNAs [83,84] and miRNAs [82] prompted us to investigate, for these cargos, the possible capability of other calix[4]arene derivatives as vectors (Figure 1), these having been previously prepared and tested in the transfection of cells with plasmid DNA [81,86,87,88,89]. All of these compounds (Figure 1) are characterized by the presence of cationic groups and of the macrocyclic hydrophobic skeleton, defined by the four aromatic rings constituting the common calix[4]arene platform; however, on the other hand, they show distinct differences in their structure. **Lys-Hex** [81] has L-lysine amino acid instead of L-arginine, with the main difference consisting of the presentation of an ammonium NH_3_^+^ group in place of the guanidinium (Gu) at the end of the lateral chain; in the delivery of plasmid DNA, this compound was found to be much less efficient than **Arg-Hex** and only in the presence of DOPE as an adjuvant did it show percentages of transfection around half of those observed with the arginine containing derivative without adjuvant. Three of the considered vectors, **Gu-Oct**, **Gu-Hex**, and **Gu-Prop**, present simple guanidinium units directly linked to the aromatic rings of the macrocyclic scaffold [86]. Previous investigation of transfection with plasmid DNA demonstrated their poor efficiency as vectors, much lower not only when compared with the lead compound **Arg-Hex**, but also with respect to **H-PropGu** [87] which, having cationic head groups at end of a C_3_ alkyl chain at the lower rim and a sufficient amphiphilic character related to the macrocyclic aromatic skeleton, evidenced significant activity in the plasmid transport with DOPE as an adjuvant. This activity, on the contrary, was completely lost in the analog **H-HexGu**, which has the guanidinium head units six methylene groups away from the macrocycle [88].

As recently done for the plasmid DNA delivery [89], we wanted to investigate the structure activity relationship for these derivatives in the transport of miRNAs and PNAs. The short length of miRNAs and, even more, the completely different backbone of PNAs with respect to plasmid DNA, lacking anionic phosphate and ribose units compared to nucleic acids, could in fact exhibit unexpected and completely different results from those observed in the DNA delivery. To this series of calixarene derivatives, we also added compound **GuPent-Hex** [90], which represents the analogue of **Arg-Hex** but lacks the ammonium group in the α position and, as a result, the stereogenic carbon atom. Its behaviour as a DNA vector has never been studied, but another calix[4]arene-based derivative, differing only in the presence of a butanoyl spacer instead of a pentanoyl one (between the guanidinium and the aromatic ring), showed very weak transfection activity [89].

### 3.2. Employment of Calix[4]arene Vectors for the Delivery of antimiRNA Peptide Nucleic Acids (PNAs)

#### 3.2.1. Assessment of the Optimal Transfection Conditions

**Figure 2:**
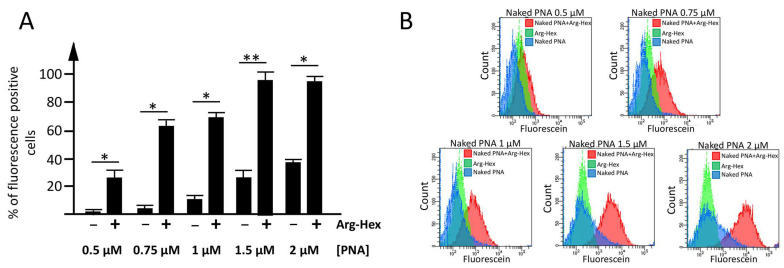
Set-up of the optimal experimental condition for the delivery of a naked PNA mediated by calix[4]arene vectors. (**A**) Histograms indicating the percentage of fluorescent positive cells, detected by FACS analysis in the absence (−) or presence (+) of the **Arg-Hex** vector, versus the increasing concentrations (0.5–2 µM) of carboxyfluorescein-labelled naked PNA. Results are presented as mean ± S.E.M; statistical differences between groups were compared using a paired *t*-test ANOVA. (*): *p* < 0.05 (significant); (**): *p* < 0.01 (highly significant). (**B**) FACS analysis plot: overlay of naked-PNA complexed with **Arg-Hex** (red), vector: **Arg-Hex** (green), naked PNA (blue).

In order to identify the optimal concentration of PNA to be delivered with calix[4]arene vectors, preliminary experiments using increasing concentrations of a carboxyfluorescein-labelled antimiR-221 PNA without a polyarginine tail (defined as ‘naked PNA’ in both, text and figures) were performed. For this series of experiments, the reference compound **Arg-Hex** (Figure 1) was employed as a vector, at the already established optimal concentration of 2.5 µM [82,83]. The human glioma cell line U251 was selected as the model system to run the set-up experiments, considering the high availability of information on its transfection conditions as reported in Gasparello et al. [82,83]. This set of the experiments allowed us to identify the final PNA concentration of 1.5 µM and the vector concentration of 2.5 µM as optimal conditions to compare the delivery efficiency (Figure 2).

Moreover, control experiments to evaluate the percentage of naked PNA internalized by cells, despite the absence of a cargo, were performed (Figure 2 and Supplementary Figure S1). Despite the absence of a vector, a low, but not negligible percentage of naked PNA enters the cells; for this reason, the data will be discussed as the difference between the cells transfected with only naked PNA and the cells transfected with the naked PNA complexed with the vector.

#### 3.2.2. Employment of Calix[4]arene Vectors for the Delivery of PNA

**Figure 3:**
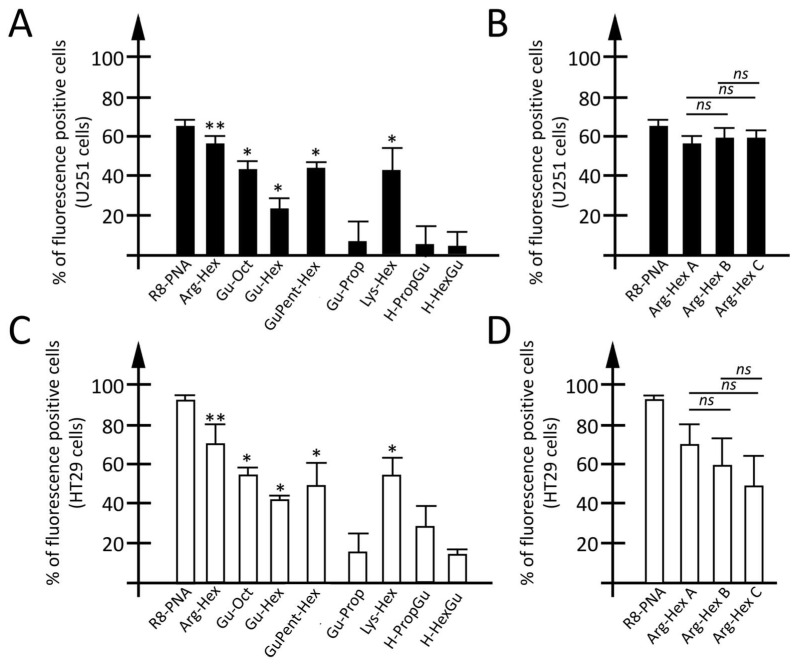
Evaluation of calix[4]arene vectors efficiency for the delivery of PNA. (**A**,**C**) Percentage of fluorescence positive cells in U251 glioma (**A**) or HT29 colon cancer cell line (**C**) transfected with an octaarginine functionalized PNA (R8-PNA) or unfunctionalized PNA (naked PNA) used alone or delivered by different calix[4]arene vectors. (**B**,**D**) Percentage of fluorescence-positive cells obtained comparing three different batches (**Arg-Hex A**–**C**) of the same vector (the lead compound **Arg-Hex**) obtained by three different purification strategies. Results are resented as mean ± S.E.M; statistical differences between groups were compared using a one-way repeated-measures ANOVA. * *p* < 0.05, ** *p* < 0.01.

The percentage of PNA transfected to the cells was determined using a fluorescent PNA and a FACS analysis, 24 h after the transfection. The carboxyfluorescein-labelled antimiR-221 PNA (defined as ‘naked PNA’ in both text and figures) was also employed to verify the ability of the other vectors to deliver PNA within cells. As positive transfection control, the carboxyfluorescein-labelled octaarginine-PNA (R8-PNA) was exploited. Previously reported data demonstrated that these R8-modified PNAs are extremely efficient in being rapidly (within hours) internalized within the cells [91,92]. Histograms reporting the percentage of fluorescent positive cells, in the case of U251 (Figure 3A,B) and HT29 (Figure 3C,D), are reported. While the fluorescence derived from vectors was negligible (0.6–10% and 0.2–4%, for U251 and HT29 respectively), the fluorescence derived from the use of the naked PNA was higher and depended to some extent on the analyzed cell line (Supplementary Figure S2). Less than 5% of fluorescent cells was detected when the HT29 cells were analyzed, while a higher (30%) naked PNA intake was found for the U251 cells. Plots reporting the percentage of fluorescence-positive cells subtracted by (a) fluorescence derived from the vector (despite being near to 0% in most of cases) and (b) the fluorescence due to the unaided naked PNA intake are presented in Figure 3. Additionally, representative plots of the FACS analysis are summarized in Supplementary Figure S3. As regards the U251 cell line, after the employment of **Arg-Hex**, about 55% of the cells were observed to be transfected when the naked PNA intake and fluorescence due to the vector were subtracted. Slightly lower was the transfection efficiency for **Lys-Hex**, **Gu-Oct** and **GuPent-Hex**, with net values of fluorescence positive cells of at least 40%. The transfection efficiency was significantly reduced when passing from **Gu-Oct** to **Gu-Hex** and **Gu-Prop**, indicating that the reduction of lipophilic chain reduces PNA delivery, even if not so dramatically as will be reported for premiRNA molecules (*vide infra*). A very low transfection efficiency was reported for **H-PropGu** and **H-HexGu**, which have the guanidinium groups at their lower rim. When colon cancer cell line HT29 was used as a cellular model, a higher transfection efficiency (around 70%) was found for **Arg-Hex**, compared with U251 cells. A similar transfection efficiency was found for the three vectors **Lys-Hex**, **GuPent-Hex** and **Gu-Oct** (ranging from 50 to 55%). Moreover, when a comparison between **Gu-Oct** and its analog **Gu-Hex** is performed, a limited difference (about 10%) in transfection efficiency is reported.

Despite being lower than that reported for the reference vector **Arg-Hex** and for **Lys-Hex**, **GuPent-Hex**, and **Gu-Oct**, a transfection efficiency of around 30% was found for **H-PropGu**, while very limited transfection efficiency was reported for **H-HexGu** (lower than 20%) and **Gu-Prop** (about 20% of transfected cells). 

Moreover, for the lead compound **Arg-Hex**, three different batches (**Arg-Hex A**, **Arg-Hex B**, and **Arg-Hex C**) were compared (Figure 3B,D) in order to verify the reproducibility of the transfection properties despite small differences having occurred in their preparation (see Supplementary Materials). While no discrepancies were found between the three vector batches in U251 cells, some differences were found between **Arg-Hex A** and **Arg-Hex B** (10%) and **Arg-Hex C** (20%) in HT29 cells.

Comparing the two cell lines (U251 and HT29), a high concordance was found for **Arg-Hex**, **Gu-Oct**, **Lys-Hex** and **GuPent-Hex**, which present high transfection efficiency in both cell lines, with the lead compound **Arg-Hex** showing the highest transfection efficiency in both cellular models. In both cell lines the **Gu-Hex** vector had lower transfection efficiency compared with the analog **Gu-Oct**. At the same time, the vectors **Gu-Prop**, **H-HexGu** and **H-PropGu** presented a low transfection efficiency in both cellular models, with **H-PropGu** showing an even lower transfection efficiency in U251, compared with HT29.

**Figure 4:**
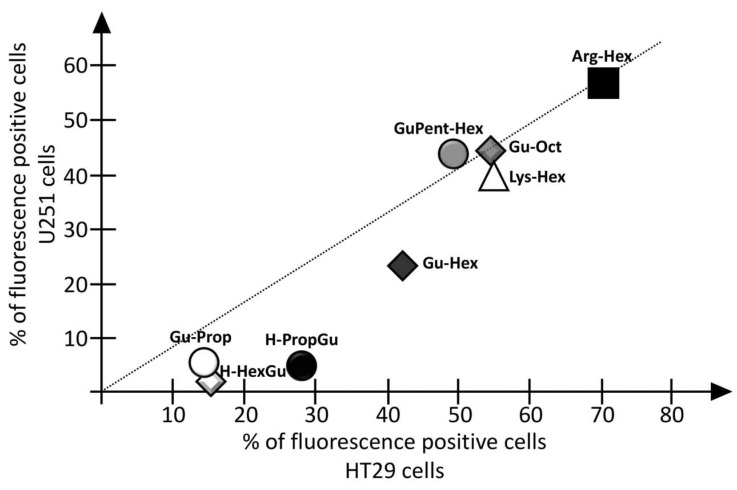
Correlation between the two cell lines transfected with calix[4]arene vectors for the delivery of PNA.

Figure 4 reports the correlation between the PNA transfection efficiency (indicated as % of fluorescence-positive cells) of the different calix[4]arene vectors in the two selected cell lines. While **Gu-Prop**, **H-HexGu** and **H-PropGu** presented low transfection efficiency in both of the analyzed cell lines, with a slightly higher activity with HT29 cells, very similar percentages of transfected cells were found in both analyzed models for the four best vectors, **Arg-Hex**, **Gu-Oct**, **Lys-Hex**, and **GuPent-Hex**. An intermediate behavior between **Gu-Oct** and **Gu-Prop** was substantially observed for **Gu-Hex** with both cell lines.

**Figure 5:**
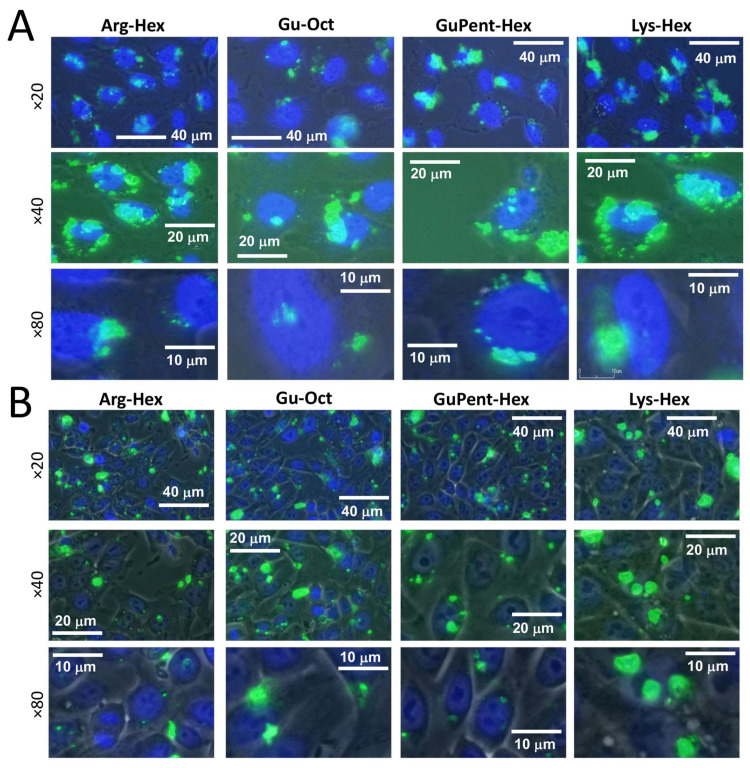
Images of fluorescein-labelled PNA delivered with calix[4]arenes. (**A**) U251 cell line, (**B**) HT29 cells. Cell nucleus is marked (dark blue) with Hoechst 33342 dye and taken using DAPI filter (461 nm), while PNA is represented by green spots and acquired using FITC filter (530 nm). Three different magnifications are reported, ×20, ×40, and ×80.

To investigate the intracellular distribution of calix[4]arene-delivered PNA, pictures of transfected cells were taken. The four most efficient calix[4]arenes, **Arg-Hex**, **Lys-Hex**, **GuPent-Hex**, and **Gu-Oct**, were selected, and the carboxyfluorescein-labelled, naked antimiR-221 PNA was delivered with them to U251 and HT29 cells. After 24 h of transfection, using the same transfection condition described for FACS analysis, the transfection medium was removed and the cells were stained with Hoechst 33342 to identify the nucleus position. Representative pictures are reported in Figure 5, from which it might be observed that the overall uptake within the cells was efficient for all of the selected vectors (additional materials are reported in Supplementary Figure S6). The fluorescence pattern in the transfected cells showed localization as dot-like structures in specific intracellular regions, particularly around the cellular nucleus. Similar distributions were previously observed for nanozeolites- [93] and for polyarginine-delivered PNAs [92], both of which have been shown to be localized into lysosomes derived from the endosomal route [93,94,95], and a similar distribution is likely to be present in this case.

### 3.3. Employment of Calix[4]arene Vectors for the Delivery of miRNA Molecules

**Figure 6:**
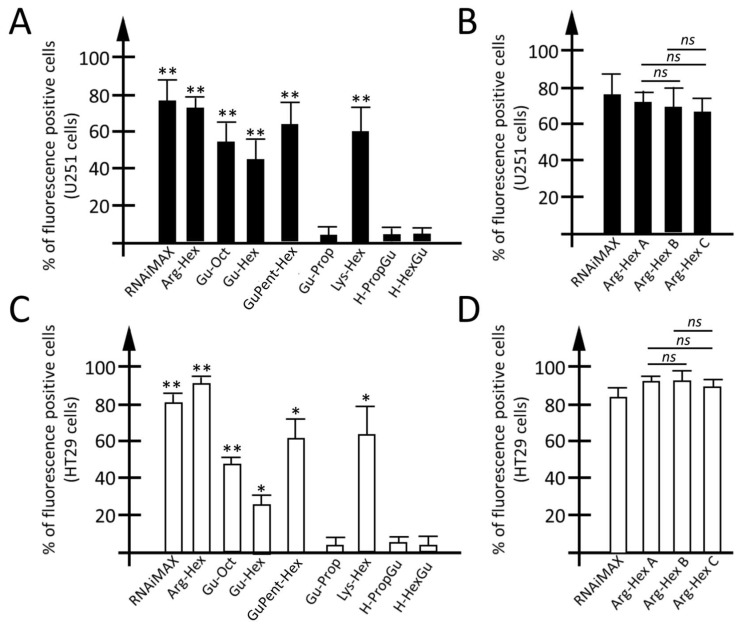
Evaluation of calix[4]arene vectors efficiency for the delivery of mature miRNA. (**A**,**C**) Percentage of fluorescence positive cells in U251 glioma (**A**) or HT29 colon cancer (**C**) cell line transfected with a mature miRNA complexed with commercially available, cationic liposomes-based transfection agent, and mature miRNA used alone or delivered by different calix[4]arene vectors. (**B**,**D**) Percentage of fluorescence-positive cells obtained comparing three different batches (**Arg-Hex A**–**C**) of the same vector, the lead compound **Arg-Hex**, obtained by three different purification strategies. Results are resented as mean ± S.E.M; statistical differences between groups were compared using a one-way repeated measures ANOVA. * *p* < 0.05, ** *p* < 0.01.

Calix[4]arene vectors were subsequently tested for their ability to deliver miRNA molecules. To this end, a synthetic sequence mimicking a mature miRNA (22 nucleotides in length) and labelled with fluorescein was employed. Thanks to the presence of fluorescein linked to the microRNA sequence, miRNA cellular internalization was verified by FACS analysis (Figure 6). As previously reported for PNAs, the vectors transfection efficiencies were verified in the two different cellular models, the glioblastoma cell line U251 and the colorectal cancer model HT29. As positive control, the mature miRNA was also transported within cells with commercially available cationic liposomes (Lipofectamine RNAiMAX). No cellular uptake was detected when the mature miRNA was administrated to cells without the use of a carrier. Representative plots derived from FACS analysis are reported for all vectors in Supplementary Figure S4. A low rate (0.6–10% for U251 cells and 0.2–4% for HT29) of fluorescent cells was found when only the vector was used. According to that found for PNA, in both U251 and HT29 cell lines (Figure 6A), a high transfection efficiency was found for the carriers **Gu-Oct**, **GuPent-Hex**, and **Lys-Hex** (ranging from 50 to 65% of fluorescence-positive cells). On the other hand, the ability of **Arg-Hex** to deliver mature miRNA (80%) was found to be comparable or even superior with respect to the commercial vector. A lower transfection efficiency was found for **Gu-Hex** (lower than 30% of fluorescent cells), while a very low or no transfection efficiency were found for the other three tested vectors. Additional data are available in Supplementary Figure S6. According to the data shown for the PNA transfection experiments, no significative differences were found when different batches (A, B, and C) of **Arg-Hex** were tested, under the same experimental conditions, in both U251 and HT29 cells (Figure 6B,D).

**Figure 7:**
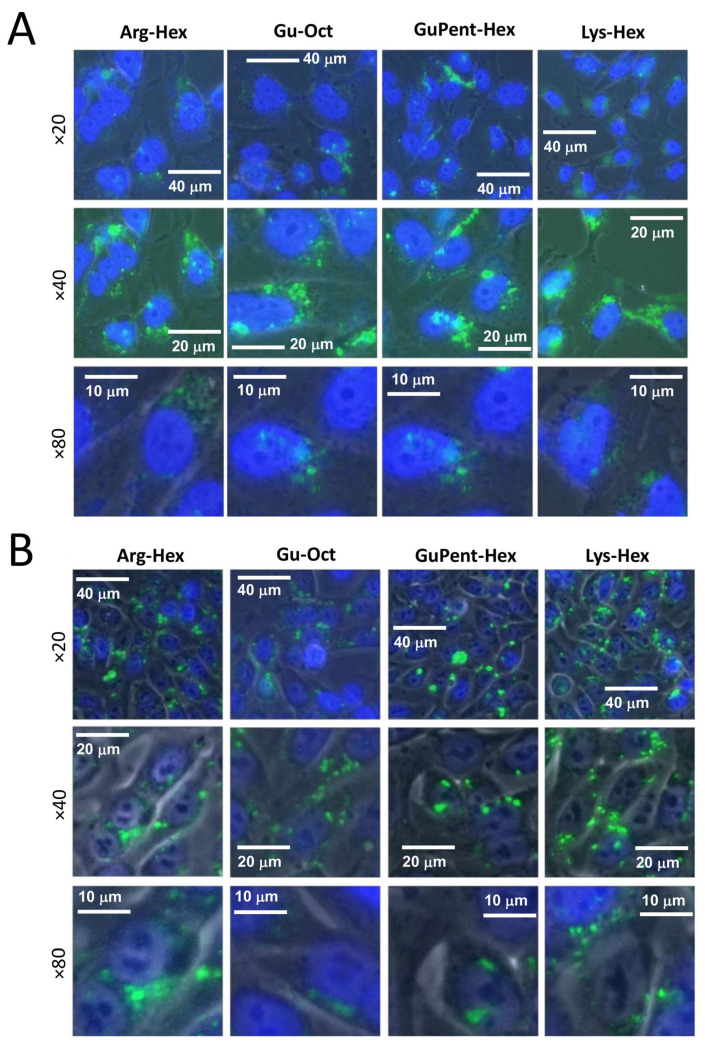
Images of fluorescence-labelled mature miRNA transfected cells. (**A**) U251 cells (**B**) HT29 cells. Cell nucleus is marked with Hoechst 33342 dye and taken using DAPI filter (461 nm), while miRNA is represented by green spots and acquired using FITC filter (530 nm). Three different magnifications are reported, ×20, ×40, and ×80.

To verify the intracellular localization of the mature miRNA delivered by the calix[4]arene carriers, pictures using a fluorescence microscope were taken. After the first preliminary screening using FACS analysis, the four most efficient vectors (**Arg-Hex**, **Lys-Hex**, **GuPent-Hex**, and **Gu-Oct**) were again chosen, and the fluorescent mature miRNA sequence, previously already employed, was delivered to the cells. After 24 h of contact with the cells, the transfection mixture was removed, and the cells washed three times and stained with Hoechst 33342 to identify nucleus position. Representative pictures are reported in Figure 7 and additional data are reported in Supplementary Figure S7. The presented pictures demonstrate the efficient uptake of the fluorescein-labelled miRNA within cells for all of the selected vectors.

### 3.4. Employment of Calix[4]arene Vectors for the Delivery of premiRNA Molecules

On the basis of the data obtained for mature miRNA delivery and considering the major complexity of the experiments used for the study of premiRNA (miRNA Precursor) molecule internalization, only the most efficient vectors for mature miRNA delivery were selected to test the transfection efficiency of premiRNA molecules. Moreover, one of the less-efficient vectors, **Gu-Prop**, was also tested as negative control. To exclude significant differences between the vectors’ transfection efficiency of mature miRNA and premiRNA, the two least-efficient **H-HexGu** and **H-PropGu** carriers were tested on the U251 cell line, indeed also confirming the low transfection efficiency for premiRNA molecules delivery. U251 glioma and HT29 colon cancer cell lines were transfected with miR-221-3p precursor (premiR-221-3p) for 24 h using calix[4]arene vectors. Briefly, premiR-221-3p was incubated for 20 min at a final concentration of 100 nM, with 2.5 µM of each vector, according to previously reported experiments by Gasparello et al. [82,83].

According to the already assessed protocol, premiRNA−vector interaction was performed in the presence of culture medium (DMEM for U251 and RPMI for HT29) without serum, which was added (at the concentration of 10%, *v*/*v*) at the end of the 20 min incubation. Twenty-four hours after the transfection, the RNA was extracted and intracellular levels of the mature miR-221-3p, derived from miR-221-3p precursor processing, were quantified through RT-qPCR. The two cell lines were selected for their similarity in physiological miR-221-3p intracellular content. 

**Figure 8:**
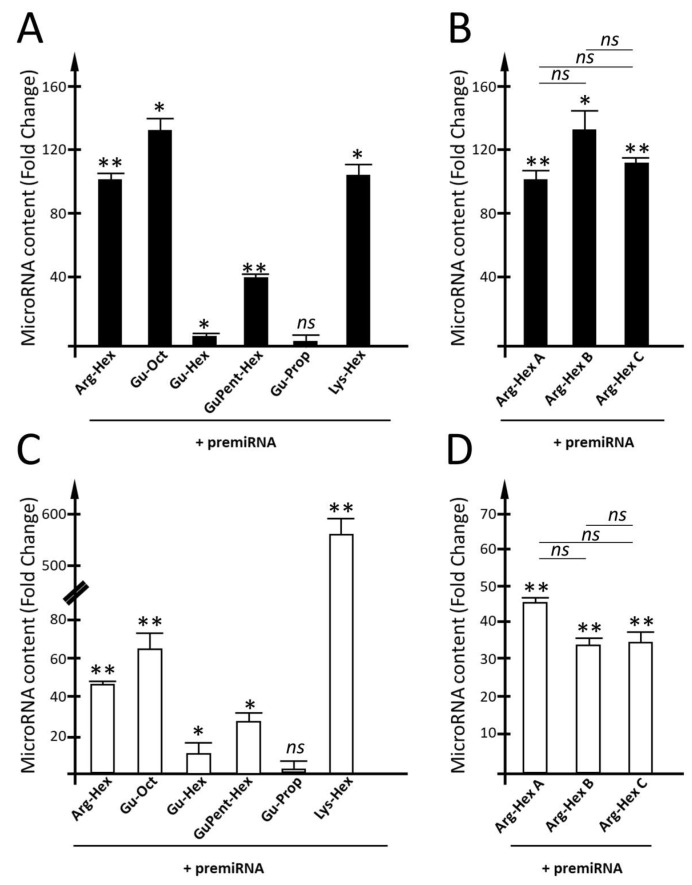
Evaluation of calix[4]arene vectors’ efficiency for the delivery of premiRNA molecules. (**A**,**B**) Quantification of miR-221-3p intracellular content within U251 cells after the transfection of premiR-221-3p delivered by calix[4]arene vectors. For each vector the fold change was calculated with respect to cells treated with the vector, not complexed with premiRNA. (**C**,**D**) Quantification of miR-221-3p intracellular content within HT29 cells after the transfection of premiR-221-3p carried by calix[4]arene vectors. Quantification of miR-221-3p has been performed by RT-qPCR. Fold change with respect to the cells treated with only the vector was calculated. Results are presented as mean ± S.E.M; statistical differences between groups were compared using a one-way repeated-measures ANOVA. * *p* < 0.05, ** *p* < 0.01.

The results of the efficiency of calix[4]arene vectors for the delivery of premiRNA molecules in the two cell line models are reported in Figure 8.

With U251 cells (Figure 8A), a high transfection efficiency was found for **Arg-Hex** and, similarly, for **Gu-Oct** and **Lys-Hex**, for which fold increases in miR-221-3p in the order of 90–130 were found. Particularly interesting is the case of **Gu-Hex**, which is similar in chemical structure to the **Gu-Oct** vector. In this case, the reduction in the lipophilic chain length is associated with a dramatic break-point in premiRNA-transfection efficiency, which was not so evident for the PNAs and mature miRNAs. A good transfection efficiency was found for **GuPent-Hex**, despite being lower than that detected for **Arg-Hex**, **Lys-Hex**, and **Gu-Oct**. A very low increase in the intracellular levels of miR-221-3p was also detected when **Gu-Prop** was used (Figure 8A). The three different batches (A, B and C) of **Arg-Hex** were compared under the same experimental conditions (Figure 8B,C). No differences were found when the two batches, A and C, were compared, while low differences, however not significant, were found when batch B was compared with batch A and batch C.

**Figure 9:**
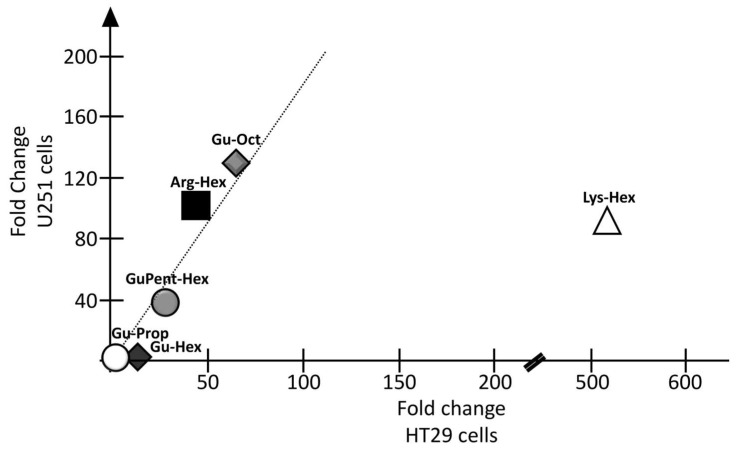
Correlation between the two cell lines transfected with calix[4]arene vectors for the delivery of premiRNA. In X axis, fold changes (FCs) calculated for premiRNA transfection in HT29 cells was reported, while FCs obtained in U251 transfected cells are reported in Y axis.

As regards HT29 cells, the transfection efficiency for **Arg-Hex** was lower than that reported in U251 cells. Even in the case of HT29 cells, a clear reduction in transfection efficiency was found when **Gu-Hex** was compared with its analog **Gu-Oct**. Again, **Gu-Prop** was found to be inactive as a vector for premiRNA delivery. Comparing the two cell lines U251 and HT29, very similar transfection efficiencies were found for **Arg-Hex**, **Gu-Hex**, **GuPent-Hex**, and **Gu-Prop** (Figure 9). A substantial difference was reported, on the contrary, for **Lys-Hex**, which, despite a good transfection efficiency in U251, was much more efficient in HT29.

**Figure 10:**
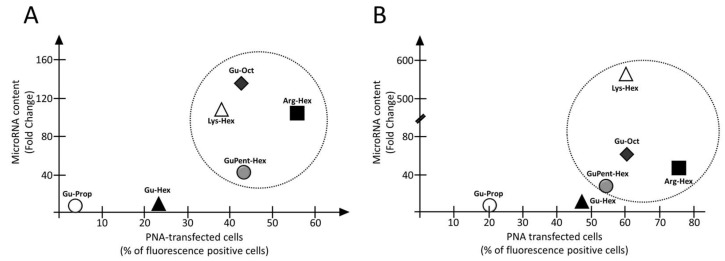
Correlation between calix[4]arene vectors efficiency for the delivery of PNA and premiRNA. (**A**) Correlation for U251 glioma cell line, (**B**) correlation for HT29 cells. Y axis transfection efficiency for premiRNA delivery expressed as MicroRNA fold change with respect to cells treated only with the vector. X axis transfection efficiency for PNA expressed as percentage of fluorescence positive cells.

As indicated in Figure 10, four vehicles, **Arg-Hex**, **Lys-Hex**, **GuPent-Hex**, and **Gu-Oct**, were found to be the best candidates for the delivery of both PNA and premiRNA molecules, despite the few expected differences between the two cell models.

### 3.5. Evaluation of Calix[4]arene Vectors Toxicity

**Figure 11:**
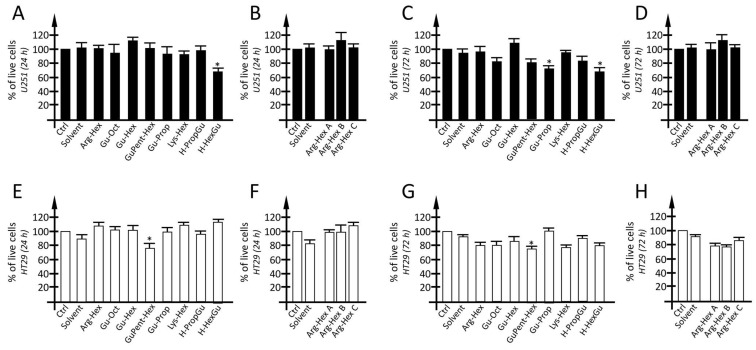
Toxicity profile of calix[4]arene vectors. Toxicity profile of glioma U251 cells (**A**–**D**) and HT29 colon cancer cells (**E**–**H**) cells after short exposure (24 h) (**A**,**B**,**E**,**F**) and middle-term exposure (72 h) (**C**,**D**,**G**,**H**) to calix[4]arene vectors. The toxicity profile was determined by MTT assay. Results are presented as mean ± S.E.M; statistical differences between groups were compared using a one-way repeated measures ANOVA. (*) *p* < 0.05, significant.

In order to verify possible cytotoxic effects deriving from the experimental conditions used in the present study, an MTT assay was carried out on U251 cells treated for 24 h (short-term treatment) and 72 h (long term treatment) with calix[4]arene vectors used at a concentration of 2.5 µM (Figure 11). The working concentration of each calix[4]arene vector was chosen on the basis of previous experiments reported in Gasparello et al. [82,83]. When U251 cells were treated with the vector working concentration for 24 h, no or very limited reductions in cell viability were detected; only for **H-HexGu** a reduction in the order of 30% was reported. Similar data were obtained when a long-term treatment (72 h) was considered: no significant reductions were observed for almost all vectors except for **H-HexGu**, for which a reduction of 25–30% in cells was detected. Regarding **H-HexGu**, the viable cell rate in the long term is comparable to that reported in the hours immediately after the treatment (24 h), indicating that no additive cytotoxic effects appear after long term exposure. Conversely, focusing on **Gu-Prop**, no cytotoxic effects were detected after a short vector exposure, while a limited reduction in cell viability was detected after prolonged exposure. A very similar toxicity profile was detected by MTT assay, when the HT29 cell line was analyzed. In particular, in the HT29 cell line, only **GuPent-Hex**, was found to present low, although significative, cytotoxic effects, both in short- and long-term treatments.

## 4. Discussion

Compound **Arg-Hex**, initially designed for plasmid DNA transport, was also subsequently found to be able to deliver into cells miRNAs and PNAs, and preserve the expected biological activity of the cargo. The use of **Arg-Hex** as a vector, for instance, for the delivery of AntimiR-221 PNA to U251 glioma cells caused a foreseen increase in apoptosis [83], while the transfection of premiR-93 was successfully associated with the significant inhibition of IL-8 and IL-6 mRNAs and a related decrease in IL-8 and IL-6 release [82]. Preliminary studies with some guanidinium calixarenes on the uptake mechanism evidenced that macropinocytosis is the most probable pathway for DNA transport across the cell membrane, being caveolae-dependent endocytosis the secondary one.

The aim of this work is to disclose the structure activity relationship for this class of calix[4]arene vectors (see Figure 1 for their chemical structure) in order to better define the boundaries of their scope, potential, and versatility. First of all, the calix[4]arene vectors display reproducible differences in their transfection activity, when both U251 and HT29 cell lines are considered. For instance, in the case of PNA delivery, **Arg-Hex**, **Gu-Oct**, **Lys-Hex** and **GuPent-Hex** exhibit the highest transfection efficiencies, similar to those displayed by PNAs functionalized with the octaarginine (R8) peptide [29,84]. Interestingly, **Gu-Hex** displays a lower transfection efficiency when compared with **Gu-Oct**. However, it is very important to underline that, despite the similar transfection efficiencies of R8-PNA and the calixarene-PNA systems, the synthesis of a R8-PNA requires undertaking a quite-demanding synthesis that also consistently lowers the final yield of the PNA conjugate. Moreover, this covalent approach should be applied to any different nucleo-base sequence on the PNA that is needed, while the use of a non-covalent vector makes these calixarene-based derivatives potentially generalizeable to any PNA.

Comparing the data of this work with the behavior showed in the past by these vectors used as delivery systems for plasmid DNA [86,87,88,89], it is interesting to underline that, while **Arg-Hex** resulted largely better regarding plasmid than all of the others, for the PNA the efficiencies of other three derivatives, **GuPent-Hex**, **Gu-Oct**, and **Lys-Hex**, are very close. This suggests that, for the transport of PNA, the absolute relevance of the simultaneous presence of guanidinium and ammonium groups, although still winning, is less binding. The guanidinium units alone, in **GuPent-Hex** and **Gu-Oct**, already confer a remarkable activity to the calix[4]arene. Analogously, the Lys units in **Lys-Hex** provide the macrocycle with an efficiency comparable to that of **Arg-Hex**, despite the absence of the guanidinium groups that are indeed essential for the delivery of DNA without adjuvants. These novel structural requirements make a larger series of options available for calixarene-mediated PNA transfection compared to DNA transfection. Relevant advantages also rise from the synthetic point of view since the vector preparation is not limited to the use of arginine, which presents some drawbacks related to secondary parasite reactions. This trend is substantially confirmed in the experiments performed on the transfection of premiRNA molecules, with **Arg-Hex**, **Lys-Hex**, and **Gu-Oct** displaying high transfection efficiency while **Gu-Pro** and **Gu-Hex** showed low transfection efficiency. In this case, the evaluation of the uptake was not based only on studies with fluorescent molecules, but also on RT-qPCR-based assays that demonstrated the differential internalization of pre-miRNA molecules upon calix[4]arene-mediated delivery, in agreement with the FACS data. Remarkably, when the FACS data were compared with the analysis using RT-qPCR-based assays amplifying mature miRNA sequences, very similar patterns were obtained with the two types of analytical approaches. This is compatible with the suggestion that the calix[4]arene vectors mediate the intracellular uptake of pre-miRNA molecules and, more importantly, do not interfere with the maturation of pre-miRNA molecules to mature miRNA, in agreement with the maintenance of biological functions of the vehiculated premiRNAs [82]. Further studies based on RT-qPCR analysis of the immunoprecipitated RISC, using co-IP, a recently described molecular approach [96], might help in clarifying the intracellular fate of the delivered pre-miRNA molecules and how much of them reach the key component responsible for their function.

Clear and general indications of this structure activity relationship study are: (i) vectors presenting guanidinium (Gu) and/or ammonium (NH_3_^+^) groups at the upper rim are much more efficient than all of the vectors having Gu groups at the lower rim; (ii) the total number of positive headgroups or the simultaneous presence of both Gu and NH_3_^+^ groups is not a very important factor since both **Lys-Hex** and **Gu-Oct** often show similar or even superior efficiency than **Arg-Hex**; (iii) the distance of the Gu groups from the calixarene scaffold is also not so important since both **GuPent-Hex** and **Gu-Oct** show remarkable transfection activity; (iv) the length of the alkyl chains is rather important as shown by a marked decrease in efficiency when going from **Gu-Oct** to **Gu-Hex** and to **Gu-Prop**. 

Interestingly, the calix[4]arene vectors essentially display the same behavior when the two cell lines (U251 and HT29) are compared, including the fact that the vectors **Gu-Prop**, **H-HexGu**, and **H-PropGu** exhibit low transfection efficiencies in both cellular models. It is interesting to note that **H-PropGu** was observed to be rather efficient in the transport of DNA when used together with DOPE as an adjuvant. However, in the present study, we opted not to use this additive because one of the desired goals regarding these calixarene-based derivatives was to have the simplest formulation possible of the cargo-vector system. After careful inspection of Figure 4 and Figure 9, it can be concluded that a similar trend of PNA and premiRNA uptake occurred, with the remarkable exception of **Lys-Hex**, which was able to deliver premiR-221 more efficiently to HT29 cells with respect to U251 cells. This result should be verified by using a larger set of target cell lines, exhibiting different rates of cell proliferation, and different membrane protein compositions, as these are factors which influence the permeability of tumor cells to external vehicles.

The most important finding of this study is that calix[4]arene-based vectors are powerful non-toxic tools to facilitate the uptake of peptide nucleic acids (PNAs) and pre-miRNA/mature miRNA molecules, which are key agents in so-called microRNA therapeutics. This proof-of-concept is, in our opinion, of great interest for the following reasons: (a) naked PNAs are known to be scarcely internalized by target cells [92]; (b) PNAs have already been demonstrated to be efficient in modifying the biological activity of target microRNAs [29,30,97,98]; (c) several delivery systems are known for premiRNA transfection, but most of them suffer from induced toxicity on target cells [38,99,100]; (d) pre-miRNAs have been extensively used to validate approaches of possible interest in personalized treatment in precision medicine.

Concerning point (b), we have already demonstrated the ability of upregulating CFTR production after the treatment of target cells with PNAs, inhibiting miR-101-3p and miR-145-5p [30,101]. This is of great interest for the development of protocols of possible interest for the clinical management of Cystic Fibrosis (CF) patients. In the case of applied oncology, PNAs targeting oncomiRNAs have already been proposed to induce phenotypic changes in target tumor cells (for instance the induction of apoptosis), thereby interfering with tumor cell growth and dissemination [33,36,102].

Concerning point (c), we show in this study that most of the calix[4]arene vectors here employed are not cytotoxic at the concentrations here used. Therefore, we expect for these vectors to be a good in vivo therapeutic window (defined as the range of drug concentrations that provide the therapeutic response without significant adverse effects). However, we cannot exclude cytotoxicity when these vectors are used at much higher concentrations. This issue will be tackled in the future, by using a large range of calix[4]arene vector concentrations. Transcriptomic and proteomic studies will be also informative and clarify this specific and very important point.

Concerning point (d), the calix[4]arene vectors may be proposed to vehiculate tumor-suppressor premiRNAs, which have been already demonstrated to be able to interfere at the transcriptional level with the expression of a high number of well-validated onco-genes. For instance, premiR-377 is used to target EGFR (Epidermal Growth Factor Receptor), which is significantly increased in NSCLC (Non-Small-Cell Lung Cancer), while mimics of miR-34a are useful to target p53 in several solid tumors [103]. Moreover, precursors of miR-181d-5p and miR-409-3p may be useful to reduce the levels of MGMT (O-6-methylguanine DNA methyltransferase) involved in temozolomide response in glioblastoma [104]. Another field of intensive study is anti-inflammatory miRNAs, which could be considered for calix[4]arene-based delivery. For instance, Fabbri et al. studied a possible link between microRNA expression and IL-8 induction in bronchial epithelial cells infected with *P. aeruginosa* and found that IL-8 protein expression is post-transcriptionally regulated by interactions of the IL-8 mRNA with the inhibitory miR-93-5p [105]. This was fully confirmed by a more recent study by Gasparello et al., who found that the production of IL-8 protein is enhanced in bronchial epithelial IB3-1 cells exposed to the SARS-CoV-2 spike protein and that IL-8 synthesis and extracellular release can be strongly reduced using a premiRNA molecule mimicking miR-93-5p [106]. The delivery of agomiRNAs-inhibiting IL-8 is of great interest for the development of therapeutic protocols for (a) down-regulating the hyperinflammatory condition of patients with Cystic Fibrosis [107,108], or (b) interfering with the COVID-19 “cytokine storm” [109,110]. Relevant to this translational issue, we would like to underline that these vectors are not toxic to the transfected cells (Figure 11) after short- (24 h) and middle-term (72 h) exposure. This allows us to propose these reagents for transfection in in vitro cell-based studies as well as for possible use in pre-clinical studies aimed to verify their suitability to be included in therapeutic protocols.

Our study suffers from some limitations. As already pointed out, only two cellular model systems have been considered. Since some differences between colorectal cancer HT29 and glioblastoma U251 cells were found, an extensive analysis of the uptake in different cell lines should be considered in the future. In addition, primary tumor cells should be considered as an informative model system. Second, no transcriptomic studies have been performed. In perspective, these would be very useful to determine whether the calix[4]arene vectors might alter the endogenous gene expression of target cells. Third, no combined treatment (i.e., co-delivery of PNAs and premiRNAs) has been considered, and no experiment was undertaken to verify possible synergism with other drugs already validated for glioblastoma and colon cancer.

In order to verify the translational impact of our study, the maintenance of the biological activity of the antagomiRNA PNA and RNA-based agomiRNA should be, in future studies, carefully analyzed. This is a highly challenging project, considering that the microRNA target of the employed anti-miR PNAs may regulate target mRNAs with the coordinated participation of other microRNAs [5,111]. Therefore, the use of single delivered PNAs may not be informative in some specific cases. In this respect, our calix[4]arene vector-based approach should consider the delivery, in addition to PNAs which target single miRNAs, of innovative PNA molecules which target multiple miRNAs. This strategy has a great impact in the treatment of cancer, as demonstrated by Wang et al. [25] who, in this context, were able to propose anti-seed PNAs which target multiple oncomiRNAs, with a potential to personalize the therapies based on tumor-specific oncomiRNAs.

Concerning the delivery of pre-miRNAs, it is expected that they can regulate different mRNA targets [5,108], causing the design of informative protocols to verify the maintenance of the biological functions of calix[4]arene-delivered agomiRNAs to be highly complex.

Another important point to clarify in the future is the structure of the vector/cargo complexes as previously done for calixarene/plasmid species [81,86,87,88,89]. Several techniques, such as Atomic Force Microscopy, Trasmission Electron Microscopy, Small-angle X-ray scattering, and fluorescence spectroscopy, even supported by molecular modelling calculations, will help in a more in-depth analysis of this aspect.

Despite these limitations, our study represents a strong proof-of-principle that calix[4]arene vectors are a class of flexible and general delivery systems for both PNAs and premiRNAs and a restricted group of them deserves to be further studied in pre-clinical settings, including the use of animal model systems.

## 5. Conclusions

The first conclusion of this study is that calix[4]arenes belong to a class of vectors that are of interest for the delivery of biomolecules to be employed in microRNA therapeutics (peptide nucleic acids based antagomiRNAs and synthetic agomiRNAs). The second conclusion is that these vectors are not toxic when used at the concentrations that allow efficient delivery. The strength of our study is its support of the concept that calix[4]arene vectors are very flexible tools for the delivery of PNAs and premiRNAs, which are molecules with very different structural features. A further important point is that our study allowed the identification of a small set of calix[4]arene vectors (**Arg-Hex** and **Gu-Oct**) which exhibit a high transfection efficiency of PNAs and miRNAs in both the studied cell lines. These two leading calix[4]arene vectors deserve, in our opinion, to be considered in further pre-clinical studies, in principle even with the co-delivery of antimiRNA PNAs and premiRNAs.

Future investigations focusing on the biological activity of the carried biomolecules (in our study PNAs and pre-miRNAs) will clarify whether the most-efficient calix[4]arenes vectors herein studied can be proposed in pre-clinical studies aimed to develop therapeutic protocols.

## Data Availability

Supplementary Materials are available. Further details and data access will be made freely available by the corresponding authors upon reasonable request.

## Supplementary Materials

The following supporting information can be downloaded at: https://www.mdpi.com/article/10.3390/pharmaceutics15082121/s1, Supplementary methods: Supplementary Method S1: calix[4]arene synthesis; Supplementary Method S2: Synthesis of PNA antimiR-221; Supplementary Method S3: Cell culture condition; Supplementary Method S4: Quantification of miRNAs intracellular content. Supplementary Figures: Supplementary Figure S1: Evaluation of naked PNA uptake; Supplementary Figure S2: Evaluation of naked PNA added with calix[4]arene vectors uptake; Supplementary Figure S3: FACS analysis plot of PNA delivery mediated by calix[4]arene vectors; Supplementary Figure S4: Evaluation of mature miRNA added with calix[4]arene vectors uptake; Supplementary Figure S5: FACS analysis plot of mature miRNA delivery mediated by calix[4]arene vectors; Supplementary Figure S6: Pictures of PNA delivery mediated by calix[4]arene vectors; Supplementary Figure S7: Pictures of mature miRNA delivery mediated by calix[4]arene vectors.
