# Supplementary material for: Cationic Calix[4]arene Vectors to Efficiently Deliver AntimiRNA Peptide Nucleic Acids (PNAs) and miRNA Mimics
Source: Pharmaceutics. 2023 Aug 10;15(8):2121. doi: 10.3390/pharmaceutics15082121 (PMC10460053; doi:10.3390/pharmaceutics15082121)
Supplement: Supplementary file 1 [file pharmaceutics-15-02121-s001.zip › pharmaceutics-2460661-supplementary.pdf]

Article

Cationic Calix[4]arene Vectors to Efficiently Deliver AntimiRNA Peptide Nucleic Acids (PNAs) and miRNA Mimics

Jessica Gasparello[1], Chiara Papi[1], Matteo Zurlo[1], Stefano Volpi[2], Roberto Gambari[1], Roberto Corradini[2], Alessandro Casnati[2], Francesco Sansone[2*] and Alessia Finotti[1, *]

[1] Section of Biochemistry and Molecular Biology, Department of Life Sciences and Biotechnology, University of Ferrara, 44121, Ferrara, Italy; gspjsc@unife.it (J.G.); chiara.papi@unife.it (C.P.); matteo.zurlo@unife.it (M.Z.); gam@unife.it (R.G.)

[2] Department of Chemistry, Life Sciences and Environmental Sustainability, University of Parma, 43124 Parma, Italy; stefano.volpi@unipr.it (S.V.); roberto.corradini@unipr.it (R.C.); alessandro.casnati@unipr.it (A.C.)

* Correspondence: alessia.finotti@unife.it (A.F.); fransesco.sansone@unipr.it (F.S.)
Tel.: +39-0532-974510 (A.F.); +39-0521-905458 (F.S.)

Supplementary materials

Supplementary methods

Supplementary Method S1. ***Synthesis of the calix[4]arene vectors***

For a detailed description of the synthesis of each vector used in this work, we refer to references reported in the manuscript bibliography.

Very briefly, the vectors bearing the positively charged groups at the upper rim (identified by the aromatic para positions of the phenolic units) of the calixarene scaffold (**Arg-Hex**, **Lys-Hex**, **Gu-Oct**, **Gu-Hex**, **Gu-Prop**, **GuPent-Hex**) were synthesized starting from the p-tert-butyl-calix[4]arene that was alkylated at the oxygen phenolic atoms with the proper chains by using NaH as base. This allows to obtain the tetraalkylated derivatives in the desired cone geometry. Subsequently, the alkylated p-tert-butyl-calix[4]arenes were nitrated at the upper rim by an ipsonitration reaction, then the nitro groups were reduced to amines. These were exploited for the coupling with protected arginine, lysine, thiourea, and aminopentanoic acid, and the following deprotection procedures gave the final derivatives.

The three different batches of the reference compound, **Arg-Hex A**, **Arg-Hex B** and **Arg-Hex C**, were obtained through synthetic procedures characterized by small differences: the arginine protected precursor of **Arg-Hex A** was synthesized in presence of dimethyl aminopyridine (DMAP) while the corresponding precursors of **Arg-Hex B** and **Arg-Hex C** in presence of diisopropyl ethyl amine (DIPEA). Moreover, the protected precursor of **Arg-Hex C** was synthesized with microwave assistance. The purification was performed for the first two by semipreparative thin layer chromatography on silica gel and for the third one by flash column chromatography on silica gel. From these protected precursors, **Arg-Hex A** was obtained by reaction in presence of TIS, while **Arg-Hex B** and **Arg-Hex C** in presence of TES. Then, the former one was firstly treated with HCl in methanol and subsequently washed with ethyl ether, the latter ones vice versa.

The vectors bearing the positively charged groups at the lower rim (identified by the oxygen phenolic atoms) of the calixarene (**H-PropGu**, **H-HexGu**) were prepared starting from the calix[4]arene by alkylation with the bromo-propyl or bromo hexylphthalimide by using NaH as base. The corresponding tetralakylated derivatives in cone geometry were obtained, then phthalimide units were removed by hydrazinolysis and the amines used for reaction with bis-Boc-triflylguanidine. The deprotection from Boc groups gave the final derivatives.

Supplementary Method S2. ***Synthesis of PNA antimiR-221***

The PNA was synthesized with standard manual Boc-based chemistry using commercially available monomers (ASM Research Chemicals, Hannover, Germany) with HBTU/DIPEA coupling. The PNA was synthesized in a 5 µmol scale using MBHA resin loaded with Boc-PNA-T-OH as first monomer. 5(6)-carboxyfluorescein (Sigma-Aldrich) was introduced using DIC/DhBtOH coupling after the coupling of the PNA.

PNA purification was performed by RP-HPLC with UV detection at 260 nm using a semi-prep column C18 (for unlabelled PNA: 5 microns, 250x10 mm, Jupiter Phenomenex, 300 Å; for fluorescein labeled PNAs: 10 microns, 300x7.7 mm, Xterra Waters, 300 Å), eluting with water containing 0.1% TFA (eluent A) and acetonitrile containing 0.1% TFA (eluent B); elution gradient: from 100% A to 50% B in 30 min, flow: 4 ml/min. Purity and identity of the purified PNAs were checked by HPLC-MS (Micromass Quattro micro API with QqQ Detector) using a Phenomenex Jupiter C18; 250x4.6 mm; 5 µm column.

Fl-PNA-a221. Yield: 27%; calculated MW: 6129,8; ESI-MS: m/z found (calculated): 1022.6 (1023.2) $[M+6H]^{6+}$, 876.8 (877.2)$[M+7H]^{7+}$, 767.4 (767.7) $[M+8H]^{8+}$, 682.2 (682.5) $[M+9H]^{9+}$, 614.0 (614.3) $[M+10H]^{10+}$.

Supplementary Method S3. ***Cell culture condition***

The human glioma U251 and the human colon cancer cell line HT29 were cultured in a humidified atmosphere of 5% CO2/air. U251 were cultured in DMEM-high glucose, additioned with L-glutamine; while

HT29 cells were maintained in RPMI-1640 medium (Euroclone). Both mediums were supplemented with 10% (v/v) fetal bovine serum (FBS; Biowest, Nuaillé, France), 100 units/mL penicillin, and 100 µg/mL streptomycin (PEN-STREP; Sigma-Aldrich, St. Louis, MO, USA).

Supplementary Method S4. ***Quantification of miRNAs intracellular content content***

For miRNA quantification, obtained RNA was reverse transcribed using a TaqMan microRNA reverse transcription kit (Applied Biosystems, Foster City, CA, USA) and specific stem-loop primers (hsa-miR-221-3p, ID: 000524). RTq-PCR was performed according to the manufacturer's protocols and as indicated elsewhere. All RT reactions, including RT-minus controls and no-template controls, were run in duplicate using the CFX96 touch real-time PCR detection system (Bio-Rad, Hercules, CA, USA). The relative expression was calculated using the comparative cycle threshold (ΔΔCT) method and hsa-let-7c (hsa-let-7c, ID: 000379) as endogenous controls.

Supplementary Figures

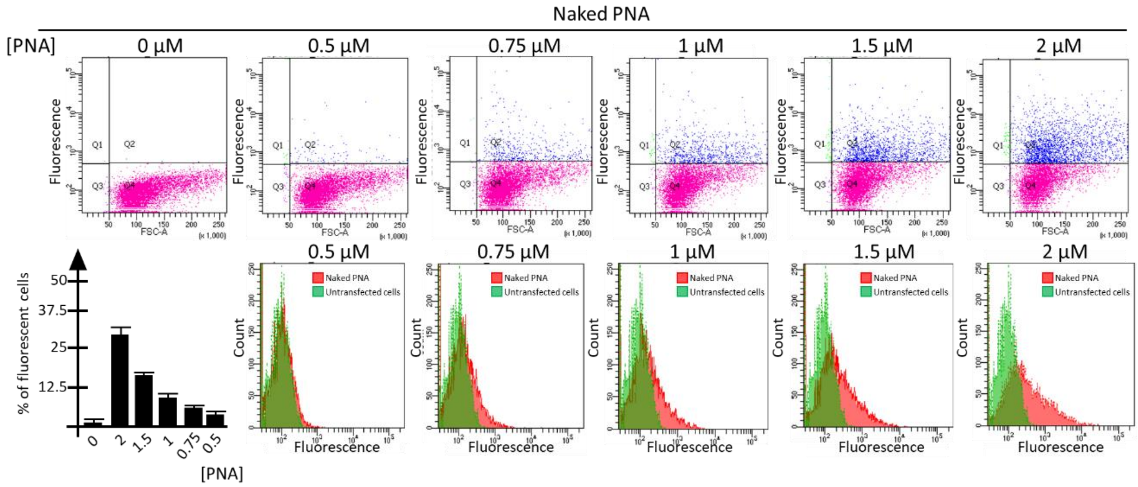

Supplementary Figure S1. Evaluation of naked PNA uptake in U251 cell line. (**A,B**) Dot plots and histograms indicating the percentage of fluorescent cells, detected by FACS analysis: blue dots: fluorescence positive cells, pink dots: fluorescence positive cells. (**C**) FACS analysis overlay: untransfected cells (green), cells transfected with naked PNA (red).

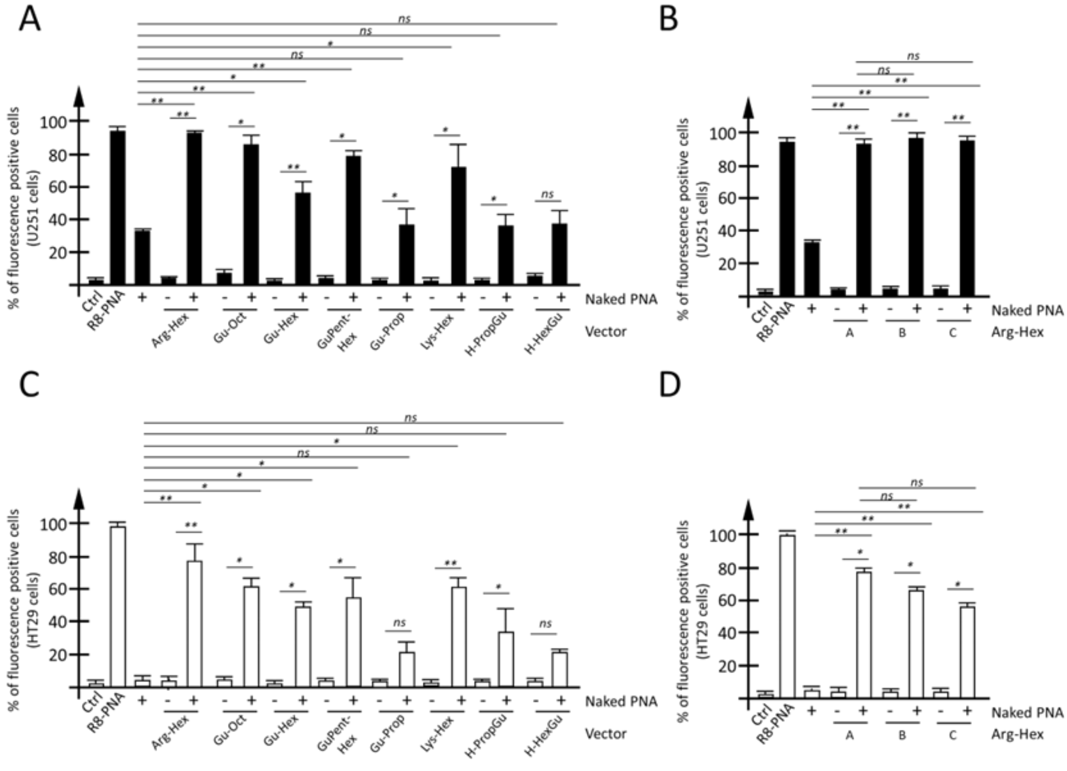

Supplementary Figure S2. Evaluation of naked PNA additioned with calix[4]arene vectors uptake.

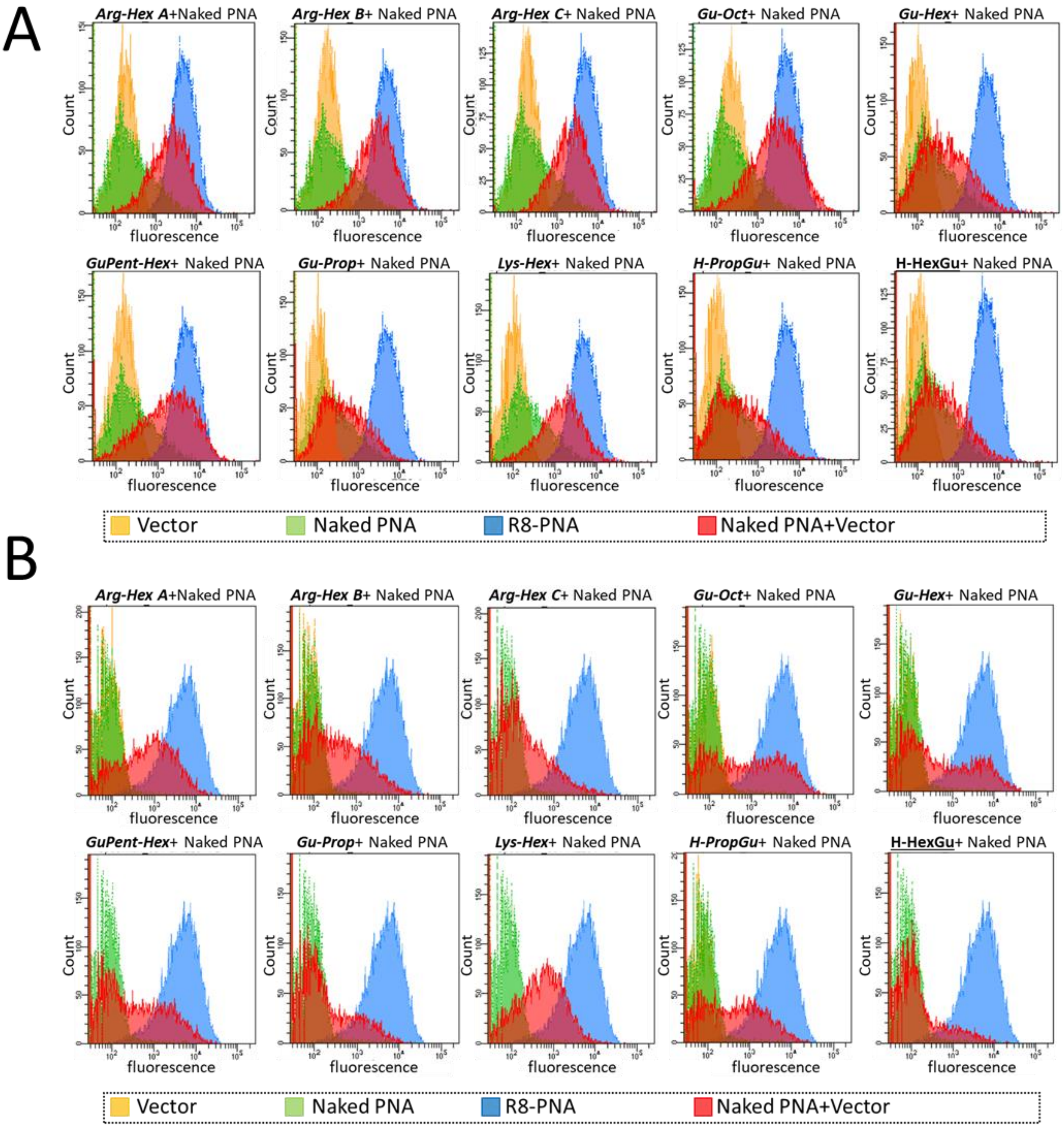

Supplementary Figure S3. FACS analysis plot of PNA delivery mediated by calix[4]arene vectors. (**A**) Plots from FACS analysis of glioma U251 transfected cells. (**B**) Plots from FACS analysis of colorectal cancer cells HT29.

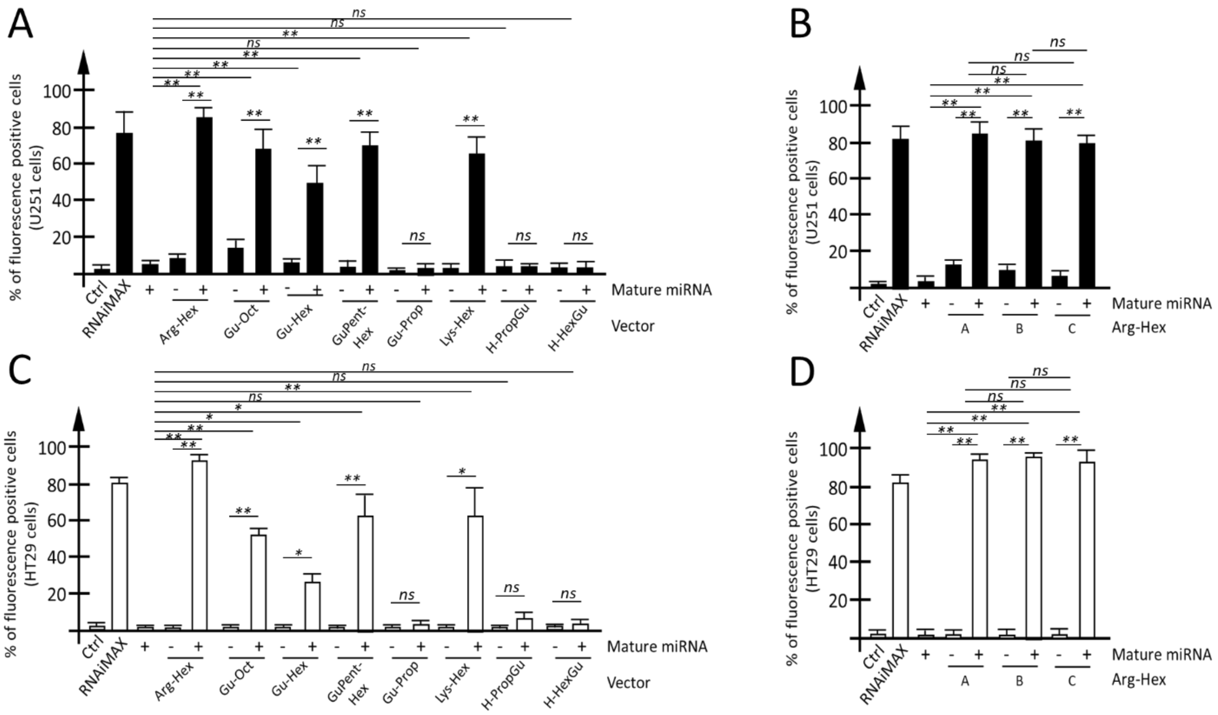

Supplementary Figure S4. Evaluation of mature miRNA additioned with calix[4]arene vectors uptake.

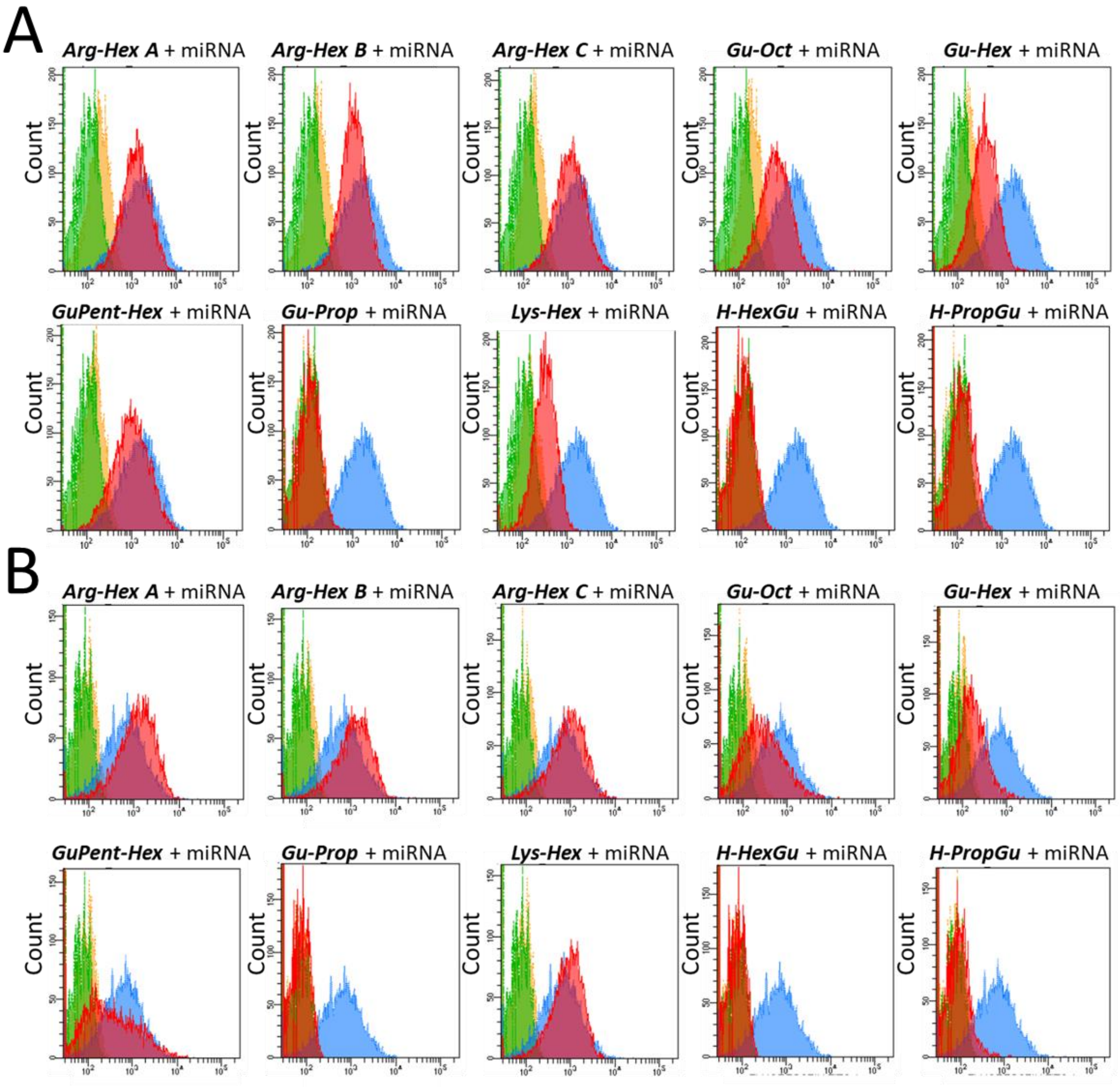

Supplementary Figure S5. FACS analysis plot of mature miRNA delivery mediated by calix[4]arene vectors. (**A**) Plots from FACS analysis of glioma U251 transfected cells. (**B**) Plots from FACS analysis of colorectal cancer cells HT29.

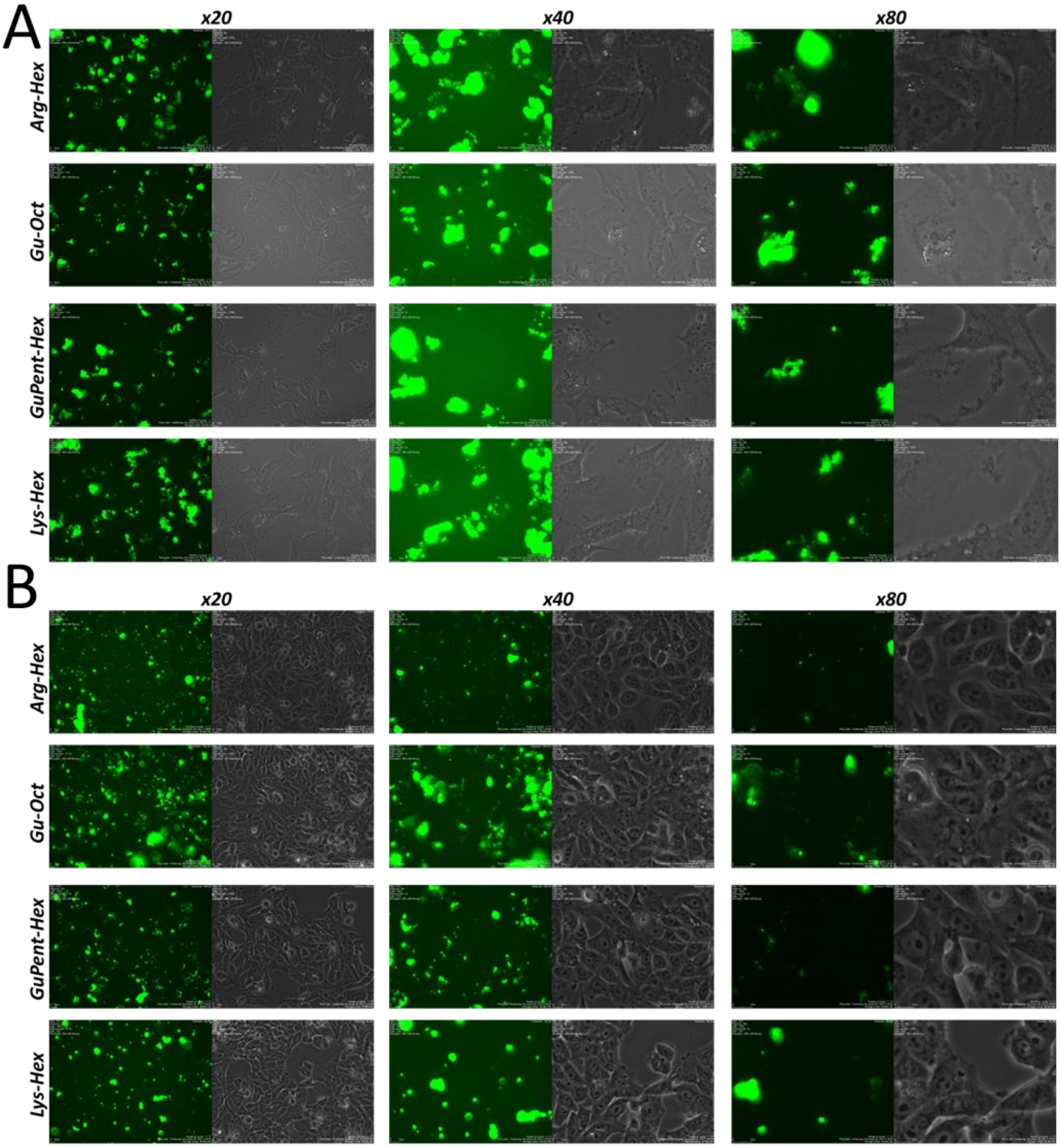

Supplementary Figure S6. Pictures of fluorescencent naked PNA delivered by calix[4]arene vectors. (**A**) U251 cell line, (**B**) HT29 cells. Pictures are taken by BioStation using FITC filter to detect PNA fluorescence.

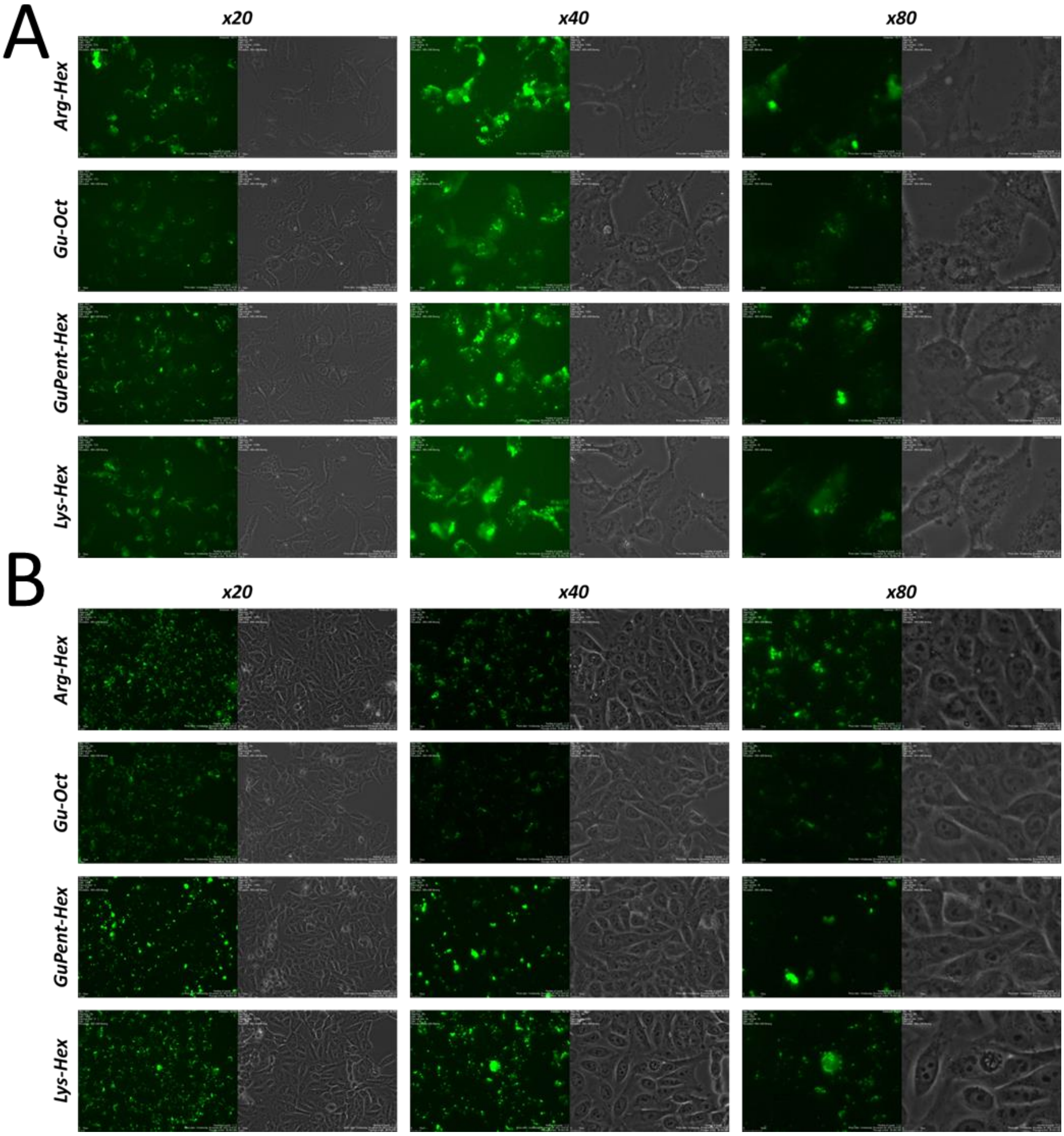

Supplementary Figure S7. Pictures of mature miRNA delivered by calix[4]arene vectors. (**A**) U251 cell line, (**B**) HT29 cells. Pictures are taken by BioStation using FITC filter to detect mature miRNA fluorescence.
